# Supplementary material for: Unusual scaling laws for plasmonic nanolasers beyond the diffraction limit
Source: Nat Commun. 2017 Dec 1;8:1889. doi: 10.1038/s41467-017-01662-6 (PMC5709497; doi:10.1038/s41467-017-01662-6)
Supplement: Supplementary file 1 — Supplementary Information [file 41467_2017_1662_MOESM1_ESM.pdf]

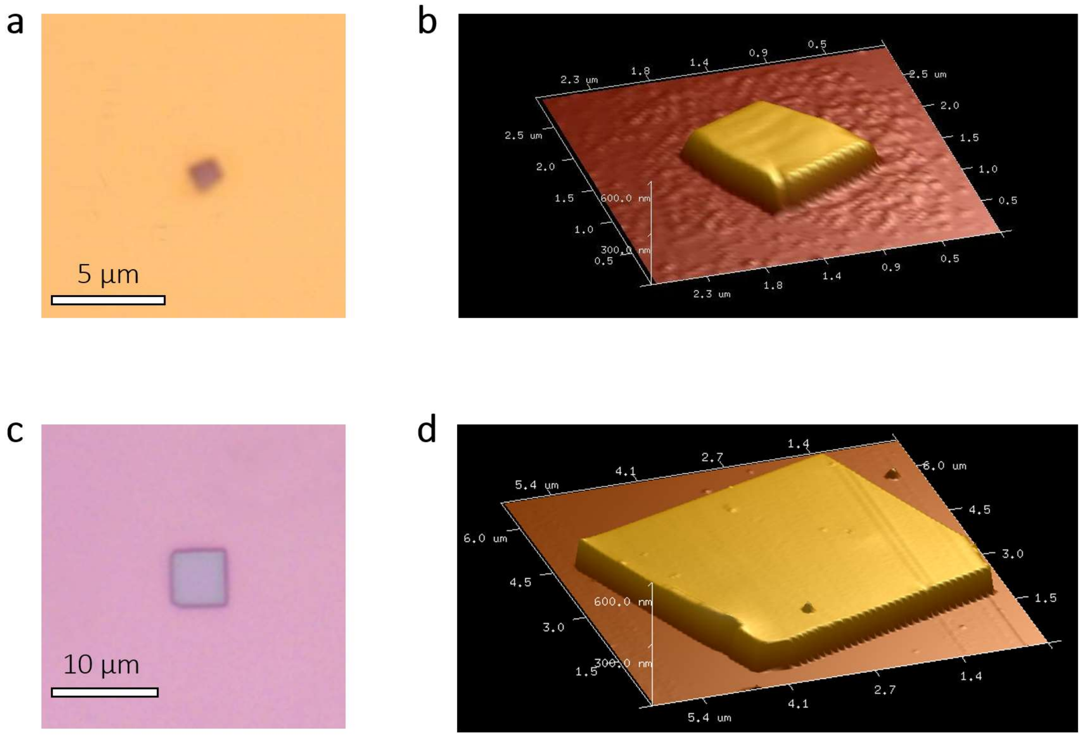

Supplementary Figure 1 | Device morphology characterization. (**a-b**) Optical (**a**) and atomic force microscope images (**b**) of a typical plasmonic nanolaser. (**c-d**) Optical (**c**) and atomic force microscope images (**d**) of a typical photonic nanolaser.

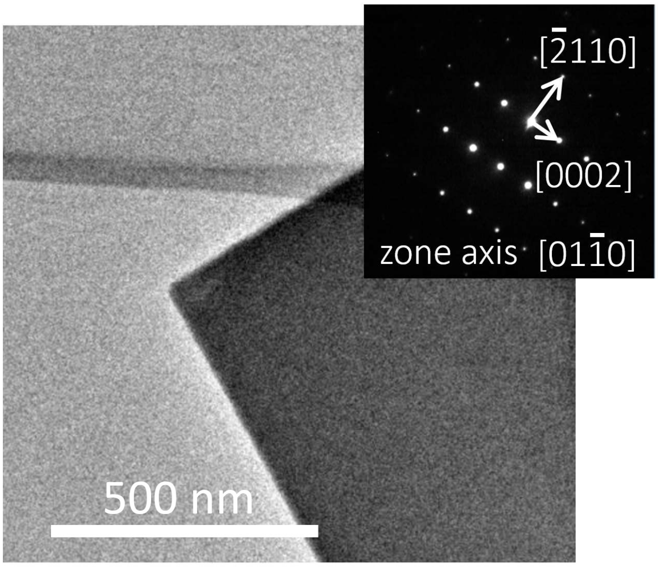

Supplementary Figure 2 | Cadmium selenide crystalline characterization. Transmission electron microscopy of a cadmium selenide nanosquare. Inset: the corresponding selected area electron diffraction pattern of the nanosquare.

Supplementary Note 1: Internal quantum efficiency of cadmium selenide nanosquares

The internal quantum efficiency (IQE) of a cadmium selenide nanosquare in a plasmonic or photonic laser cavity can be written as

$$\eta_{\mathrm{i}} = \frac{R_{\mathrm{m}}+R_{\mathrm{PF}}}{R_{\mathrm{m}}+R_{\mathrm{PF}}+R_{\mathrm{non}}} \tag{1}$$

where R_{m} is the background material radiative emission rate, R_{non} is the non-radiative emission rate and R_{PF} is the additional radiative emission rate accelerated by the Purcell effect.

The spontaneous emission lifetime of a cadmium selenide nanosquare in a plasmonic or photonic laser cavity can be written as

$$\tau = \frac{1}{R_{\mathrm{m}}+R_{\mathrm{PF}}+R_{\mathrm{non}}} \tag{2}$$

And that of cadmium selenide without cavity Purcell enhancement is,

$$\tau_{\mathrm{m}} = \frac{1}{R_{\mathrm{m}}+R_{\mathrm{non}}} \tag{3}$$

To characterize the IQE of nanosquares in the plasmonic and photonic nanolaser cavities, we measured the spontaneous emission lifetime of the large cadmium selenide nanosheets (without cavity enhancement) and the nanosquares in plasmonic and photonic nanolaser cavities (with cavity enhancement).

Supplementary Fig. 3a shows the lifetimes of a large nanosheet and nanosquares in the plasmonic and photonic cavities. The lifetime of the large cadmium selenide nanosheet is about 5.8 ns. The lifetimes of the cadmium selenide nanosquares in the plasmonic and photonic cavities are about 0.08 ns and 0.47 ns, respectively, both of which are significantly shortened by Purcell enhancement.

The lifetimes of thirteen large nanosheets are measured which gives an average lifetime of 5.6 ns for cadmium selenide without cavity enhancement as shown in supplementary Fig. 3b. According to the lifetime and its corresponding IQE of cadmium selenide reported in ref. 1, the IQE of our cadmium selenide without cavity enhancement is

calculated to be about 30%. We then calculate IQEs of nanosquares with cavity enhancement using Supplementary Equation (1) to (3). For example, the IQE of cadmium selenide nanosquares with lifetimes of 0.08 ns and 0.47 ns as shown in Supplementary Fig. 3a are about 99% and 94%, respectively which approaches to unity.

Following the same procedure, we obtain the IQEs of cadmium selenide nanosquares in all the plasmonic and photonic nanolaser cavities as shown in supplementary Fig. 3c. The Gaussian peak for plasmonic nanolasers is around 90.3% and that for photonic nanolasers is around 78.4%. Under the lasing condition, the dominant emissions are the stimulated emissions, which are with even shorter lifetimes than spontaneous emissions. So IQEs for both plasmonic and photonic nanolasers should be significantly larger than these shown in supplementary Fig. 3c.

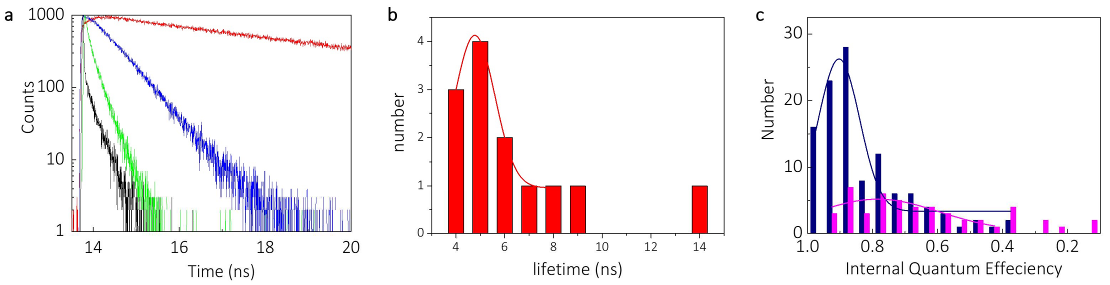

Supplementary Figure 3 | Internal quantum efficiency of cadmium selenide nanosquares. (**a**) Time-resolved spectra of large cadmium selenide sheet (red curve) and nanosquares in plasmonic (green curve) and photonic (blue curve) nanolaser cavities. The lifetime of the large cadmium selenide nanosheet is about 5.8 ns. The lifetimes of the cadmium selenide nanosquares in the plasmonic and photonic cavities are about 0.08ns and 0.47ns, respectively. Black curve: instrument response function. (**b**) Lifetime distribution histogram of large cadmium selenide sheet without cavity enhancement. Line: Gaussian distribution fitting. (**c**) IQEs distribution histogram of plasmonic (navy columns) and photonic (magenta columns) nanolasers. Lines: Gaussian distribution fittings.

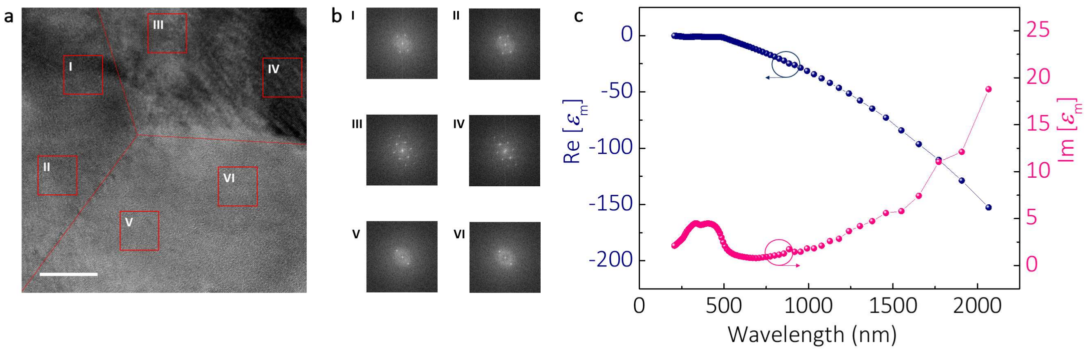

Supplementary Figure 4 | Characterization of gold polycrystalline thin film. (**a**) High resolution transmission electron microscope image of the gold polycrystalline thin film showing grain size larger than 60 nm. Scale bar: 10 nm. (**b**) Selected area electron diffraction (SAED) patterns of the marked area in (**a**). The SAED patterns of different positions in a grain share the same feature. (**c**) Permittivity of gold film (ε_{m}) measured by an ellipsometer. The quality factor ($\frac{-\mathrm{Re}[\varepsilon_{\mathrm{m}}]}{\mathrm{Im}[\varepsilon_{\mathrm{m}}]}$) of the gold film is 16 around 700 nm. Lines: guides to the eye.

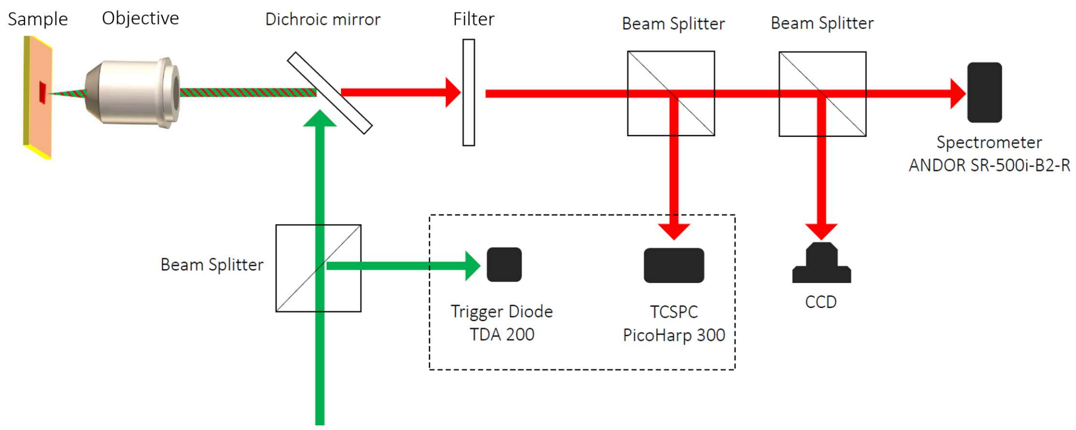

Supplementary Figure 5 | Schematic of the optical setup. The nanolasers were optically pumped by a nanosecond pump laser (λ_{pump} =532 nm, repetition rate: 1 kHz, pulse length: 4.5 ns). A 20× objective lens (NA=0.4) was used to focus the beam to a ~20 μm diameter spot on the sample surface to pump the nanolasers. The emission from the nanolasers is collected by the same objective. To obtain the time-resolved spectra, a time-correlated single photon counting system (PicoHarp 300) is employed, where a femtosecond laser is used as the pump laser (λ_{pump} = 405 nm, repetition rate: 4 MHz, pulse width: ~ 140 fs).

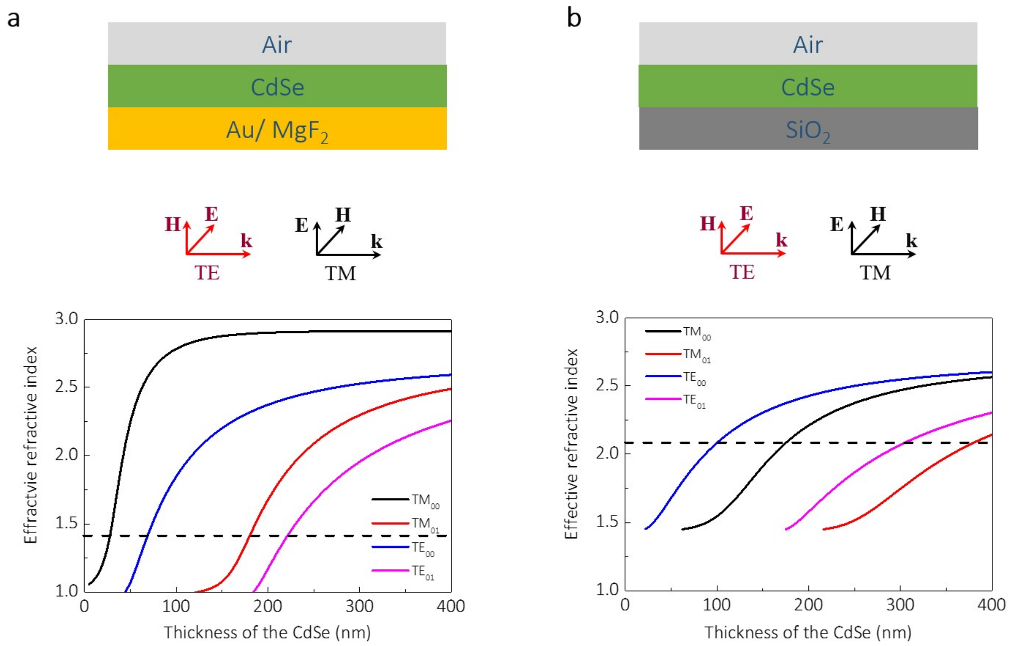

Supplementary Figure 6 | Effective refractive indices. (**a**) Effective refractive indices of the lowest four orders of waveguide modes in cadmium selenide-magnesium fluoride-gold system. (**b**) Effective refractive indices of the lowest four orders of waveguide modes in cadmium selenide-silicon dioxide system. Dashed lines in (**a**) and (**b**) indicate the lowest effective refractive indices to support a total internal reflection (TIR) cavity mode. TIR feedback in a nanosquare cavity requires a critical angle of TIR, $\theta_c \geq 45^\circ$, which requires the effective refractive index of the cavity mode, $n_c \geq \sqrt{2}\ n_s$. Here, n_s is the effective refractive index of the surrounding.

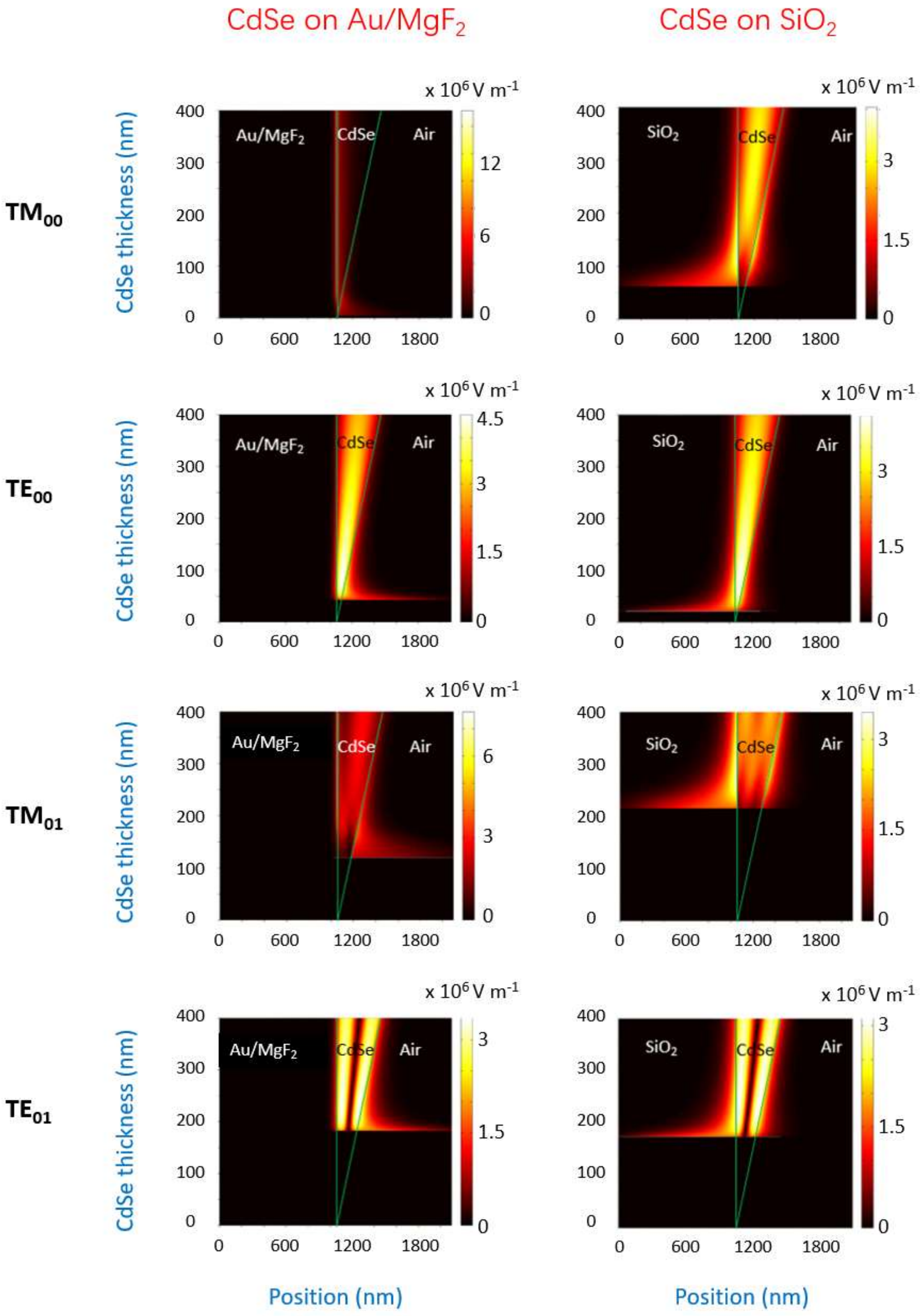

Supplementary Figure 7 | Near-field distributions. Near-field distributions for the lowest four orders of modes in cadmium selenide - magnesium fluoride - gold and cadmium selenide - silicon dioxide systems. For all the panels, x-axis indicates position in the unit of nanometre; y-axis indicates the thickness of cadmium selenide in the unit of nanometre. For each mode, we have calculated the near-field distribution for cadmium selenide thickness varied from 0 to 400 nm. The green lines in each panel are guidance for the eye to track the changing cadmium selenide thickness.

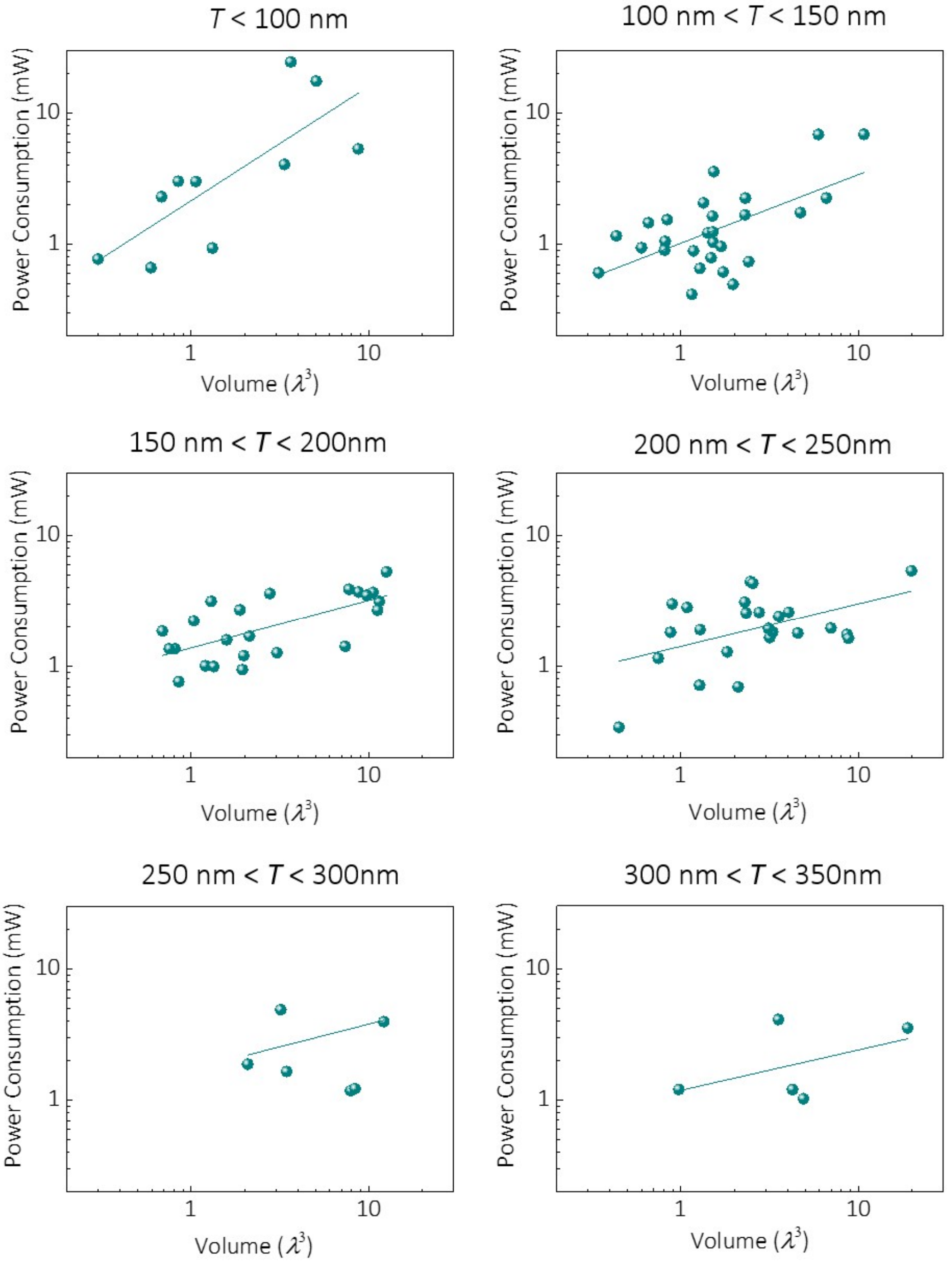

Supplementary Figure 8 | Scaling laws of power consumption versus device volume for plasmonic lasers. Lines are fitting curves with $P_{\mathrm{th}}^{(\mathrm{power})} \propto V^{\alpha}$, where $P_{\mathrm{th}}^{(\mathrm{power})}$ is the power consumption at threshold, V is the device volume and α is an exponent. The fitted α are 0.82, 0.87, 0.36, 0.32, 0.34, 0.30 for thickness ranges of T < 100 nm, 100 nm < T < 150 nm, 150 nm < T < 200 nm, 200 nm < T < 250 nm, 250 nm < T < 300 nm, 300 nm < T < 350 nm, respectively. In all panels, λ refers to 700 nm.

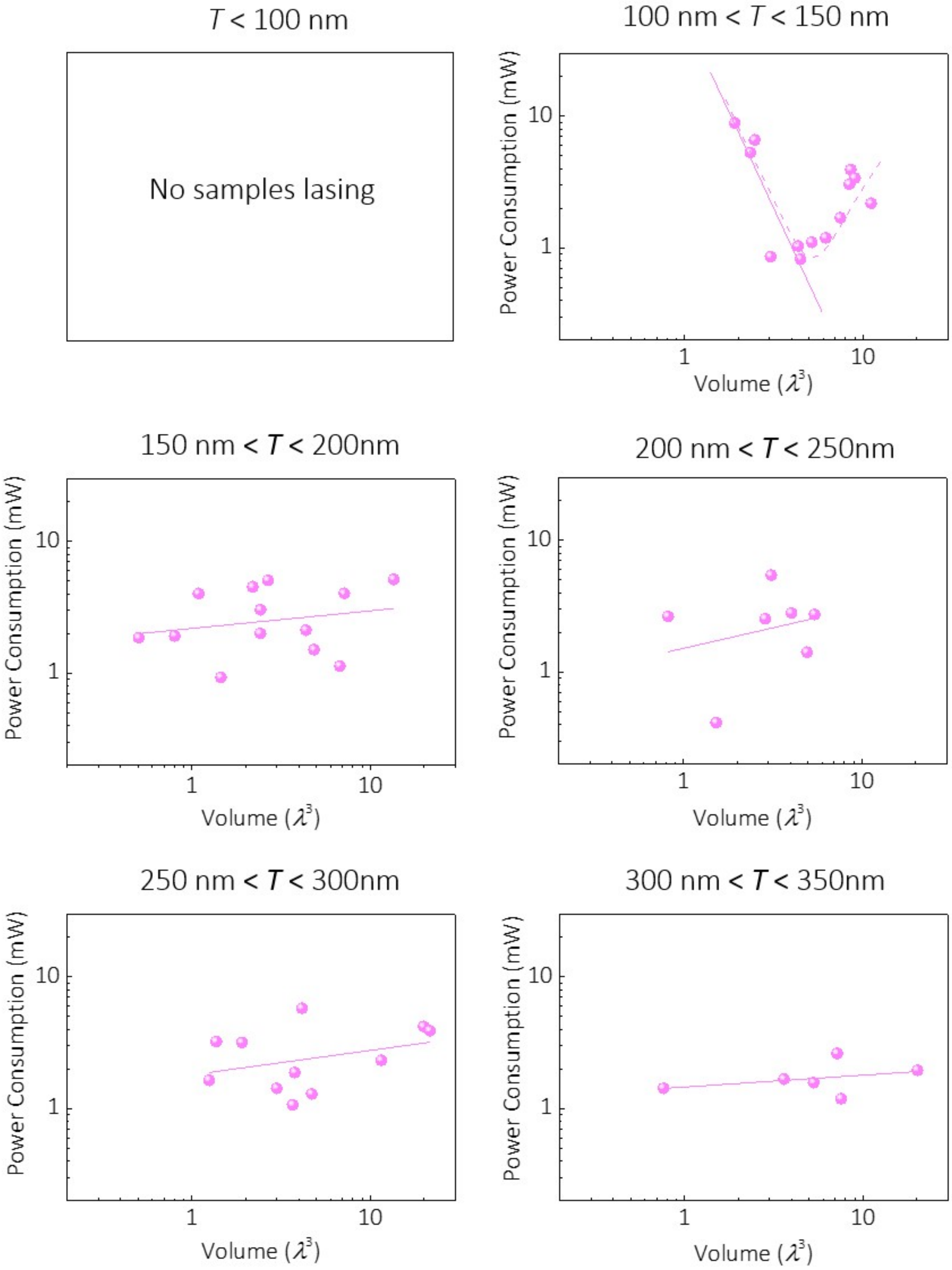

Supplementary Figure 9 | Scaling laws of power consumption versus device volume for photonic lasers. Lines are fitting curves with $P_{\mathrm{th}}^{(\mathrm{power})} \propto V^{\alpha}$, where $P_{\mathrm{th}}^{(\mathrm{power})}$ is the power consumption at threshold, V is the device volume and α is an exponent. The fitted α are -2.91, 0.13, 0.31, 0.19, 0.09 for thickness ranges of 100 nm < T < 150 nm, 150 nm < T < 200 nm, 200 nm < T < 250 nm, 250 nm < T < 300 nm, 300 nm < T < 350 nm, respectively. In all panels, λ refers to 700 nm. Dashed line: guide to the eye.

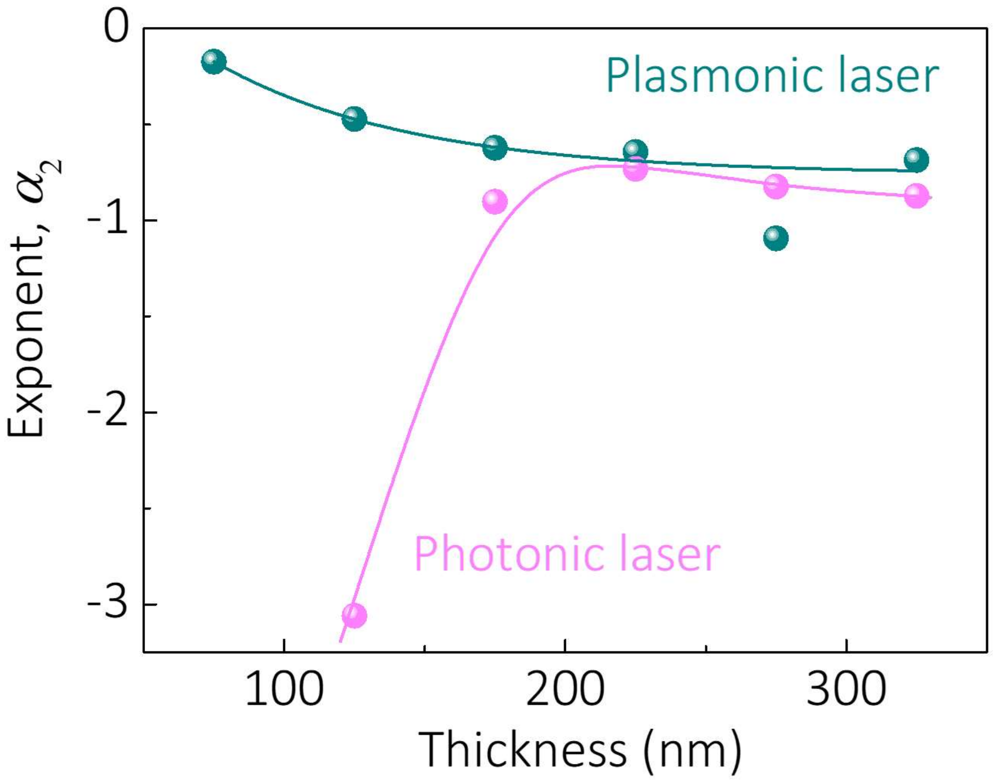

Supplementary Figure 10 | Quantitative analysis of threshold scaling of plasmonic and photonic lasers. For each range of device thickness, a phenomenological scaling law of threshold versus volume is expressed as $P_{\mathrm{th}} \propto V^{\alpha_2}$, where P_{th} is the threshold, V is the device volume and α_2 is an exponent.

Supplementary Note 2: cavity quality factor and mode volume

Cavity modelling: Numerical modelling was done using a finite-element method eigenmode solver (Comsol Multiphysics). For plasmonic nanolasers, a cadmium selenide ($n_{\text{Cadmium selenide}} = 2.8 + 0.0017i$) nanosquare lies on a gold ($\varepsilon_{\text{m}} = -16.486 + 1.0643i$) substrate separated by 5 nm magnesium fluoride ($n_{\text{MgF2}} = 1.8937$). For photonic nanolasers, the substrate is changed to silicon dioxide ($n_{\text{SiO2}} = 1.4553$). The cavity quality factor (Q) is calculated by $Q = \frac{\text{Re}(\omega)}{2\text{Im}(\omega)}$, where ω is the complex eigenfrequency. The mode volume of a cavity (V_{mode}) is calculated by, $V_{\text{mode}} = \frac{\int W_{\text{em}}(\mathbf{r})d^3\mathbf{r}}{\max[W_{\text{em}}(\mathbf{r})]}$, where $W_{\text{em}}(\mathbf{r})$ is the electromagnetic energy density of the mode. $W_{\text{em}}(\mathbf{r})$ is calculated using the formula, $\frac{1}{2}[\text{Re}\left[\frac{d(\omega\varepsilon)}{d\omega}\right]|\mathbf{E}(\mathbf{r})|^2 + \mu|\mathbf{H}(\mathbf{r})|^2]$ accounting for the dispersive electric energy density of the cavity mode in the gold. All values of Q and V_{m} here are for total internal reflection modes of TM_{00} mode and TE_{00} mode, which are with strongest field confinement and highest effective refractive index in plasmonic and photonic cavities respectively. We note that the mode volume calculated here may be overestimated due to the inherent field divergence of leaky cavity eigenmodes [2-3].

Simulation results: Supplementary Fig. 11a shows mode volume scaling laws for plasmonic and photonic laser cavities. First, the mode volume of a plasmonic cavity is about one order of magnitude smaller than photonic ones at a same physical volume. Such a fact is due to the strong plasmonic field localization effect at the metal-insulator-semiconductor interface. Second, the mode volume of a plasmonic cavity changes slowly with physical volume than photonic ones.

Supplementary Fig. 11b shows quality factor scaling laws for plasmonic and photonic laser cavities. For photonic cavities, the scaling of Q versus V can be divided into two ranges. At small V, Q is mainly determined by radiation loss and increases with V with an exponent index of 0.68. At large V, radiation loss becomes smaller, and Q is mainly limited by the self-absorption of Cadmium selenide. As a result, the growth rate of Q

with V slows down with an exponent of 0.30. For plasmonic laser cavities, Q is limited by the metallic loss and is inert to the size change. The simulation result gives an exponent of 0.1.

Supplementary Fig. 11c and d show scaling laws of $\frac{V_m}{Q}$ versus volume for photonic and plasmonic cavities, which are calculated based on Supplementary Fig. 11a and b. For photonic cavities, $\frac{V_m}{Q}$ changes with physical volume with an exponent index of 0.22 and 0.60 at small and large volume ranges respectively. For plasmonic lasers, the exponent is 0.53. The lifetime τ should be proportional to $\frac{V_m}{Q}$, so the relationship between $\frac{V_m}{Q}$ and physical volume corresponds to the scaling laws of lifetime with physical volume.

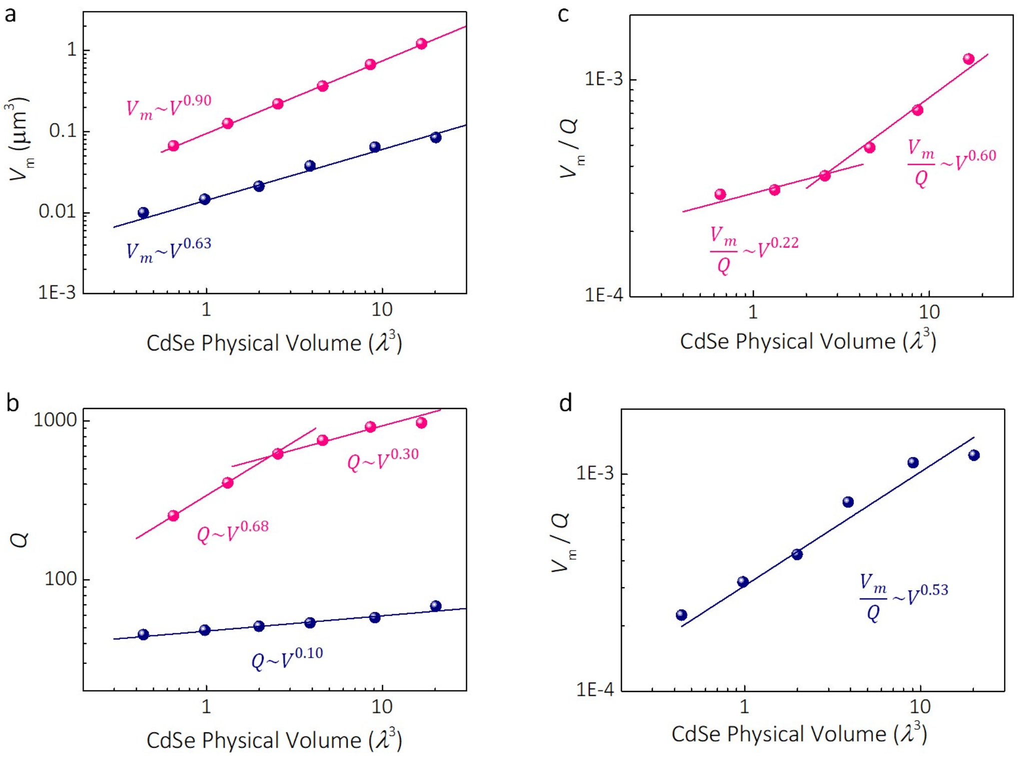

Supplementary Figure 11 | Scaling laws of quality factor and mode volume versus device volume. (**a-b**) Quality factor (Q) and mode volume (V_m) versus physical volume for plasmonic (navy dots) and photonic (pink dots) lasers cavities. Lines: exponentially fitted curves. (**c-d**) V_m/Q versus physical volume for photonic (**c**) and plasmonic (**d**) lasers cavities. In all panels, λ refers to 700 nm.

Supplementary Note 3: rate equation analysis

We first start with semiconductor rate equations that follow:

$$\frac{dN_2}{dt} = \eta p - R_{\mathrm{non}} N_2 - R N_2 - \Gamma R\ \beta (N_2 - N_0) N_{\mathrm{ph}} \tag{4}$$

$$\frac{dN_{\mathrm{ph}}}{dt} = -\gamma N_{\mathrm{ph}} + \beta R N_2 + \Gamma R\ \beta (N_2 - N_0) N_{\mathrm{ph}} \tag{5}$$

Here, N_2 is the excited carrier population, N_{ph} is the photon number of a single mode laser, p is the pump rate, N_0 is the excited state population at transparency, η is the conversion efficiency of pump photons into electron/hole pairs, R_{non} is the nonradiative recombination rate, R is the spontaneous emission rate, Γ is the confinement factor, β is the spontaneous emission coupling factor, γ is the total cavity loss rate.

The Supplementary Equation (4) describes the rate of change of the carrier population, while the Supplementary Equation (5) describes the rate of change of the photon number. In the quasi steady state conditions considered in our experiments, $\frac{dN_2}{\partial t} = 0$ and $\frac{dN_{ph}}{\partial t} = 0$, the photon number N_{ph} can be described by the quadratic equation:

$$\gamma {N_{\mathrm{ph}}}^2 - [\eta p - \frac{R_{\mathrm{non}} + R}{\Gamma R\ \beta} \gamma - (R_1 + R) N_0 + \beta R N_0] N_{\mathrm{ph}} - \frac{\eta p}{\Gamma} = 0 \tag{6}$$

The solution of the above equation has two linear trends at large and small pump rates p respectively, which define the conventional kink in the light-light curve. We define the threshold at the value of power density where these linear trends intersect. The pump power density is related to pump rate by $P = ph\nu/A$, where $h\nu$ is the photon energy and A is the device area. The threshold P_{th} is thus,

$$P_{\mathrm{th}} = \frac{h\nu}{\eta} \left[\frac{1 - \eta_{\mathrm{i}} \beta}{\eta_{\mathrm{i}} \Gamma \beta \mathrm{A}} \cdot \gamma\ + R_{\mathrm{non}} n_0 T + (1 - \beta) n_0 T \cdot \frac{1}{\tau} \right] \tag{7}$$

Here, $\eta_i = \frac{R}{R_{\mathrm{non}} + R} \approx 1$ is the internal quantum efficiency of the gain material, so that $R_{\mathrm{non}} \ll R$. $\tau \approx \frac{1}{R}$ is the spontaneous emission lifetime. $n_0 = \frac{N_0}{V}$ is the excited state

density at transparency where V is the volume of the Cadmium selenide. $T = V/S$ is the thickness of the cadmium selenide nanosquare.

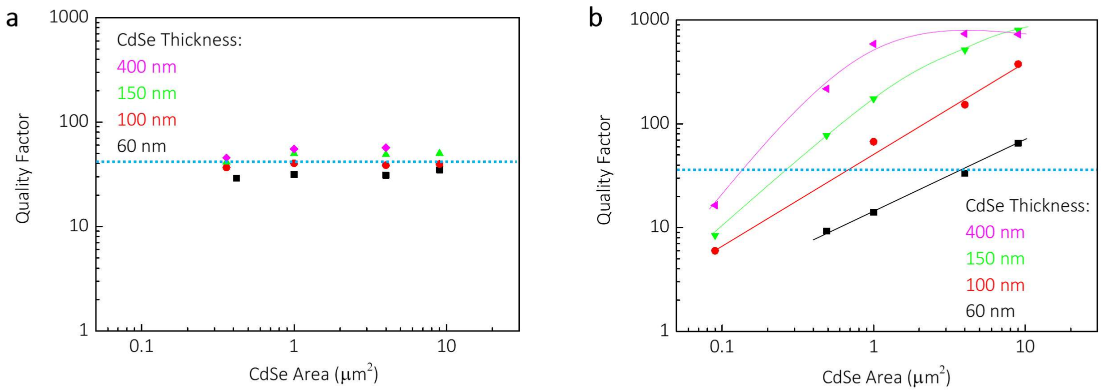

Supplementary Figure 12 | Scaling laws of quality factor with device area categorized by cadmium selenide thickness. (**a-b**) Scaling laws of quality factor with device area for plasmonic (**a**) and photonic (**b**) laser cavity modes. For each case, the quality factor is calculated for a cadmium selenide nanosquare with thickness of 60 nm, 100 nm, 150 nm and 400 nm respectively. The dotted lines in both figures indicate the same Q value. Here, all values of Q are for total internal reflection modes of TM_{00} mode and TE_{00} mode, which are with strongest field confinement and highest effective refractive index in plasmonic and photonic cavities respectively. Lines are guides to the eye.

Supplementary References

1. Shimizu. K. T., Woo. W. K., Fisher. B. R., Eisler. H. J., Bawendi. M. G. Surface-Enhanced Emission from Single Semiconductor Nanocrystals. *Phys. Rev. Lett.* **11,** 89 (2002).

2. Kristensen, P. T. & Hughes, S. Modes and mode volumes of leaky optical cavities and plasmonic nanoresonators. *ACS Photonics* **1**, 2-10 (2014).

3. Sauvan, C., Hugonin, J. P., Maksymov, I. S. & Lalanne, P. Theory of the spontaneous optical emission of nanosize photonic and plasmon resonators. *Phys. Rev. Lett.* **110**, 237401 (2013).
